# Molecularly Imprinted Wearable Sensor with Paper Microfluidics for Real-Time Sweat Biomarker Analysis

**DOI:** 10.1021/acsami.4c10033

**Published:** 2024-08-23

**Authors:** Mayank Garg, Heng Guo, Ethan Maclam, Elizabeth Zhanov, Sathwika Samudrala, Anton Pavlov, Md Saifur Rahman, Myeong Namkoong, Jennette P. Moreno, Limei Tian

**Affiliations:** † Department of Biomedical Engineering, Texas A&M University, College Station 77843, Texas, United States; ‡ Center for Remote Health Technologies and Systems, Texas A&M University, College Station 77843, Texas, United States; § Department of Pediatrics-Nutrition, Baylor College of Medicine, Houston 77030, Texas, United States

**Keywords:** wearable sweat sensors, molecularly imprinted polymer, real-time monitoring, laser-induced graphene, paper microfluidics

## Abstract

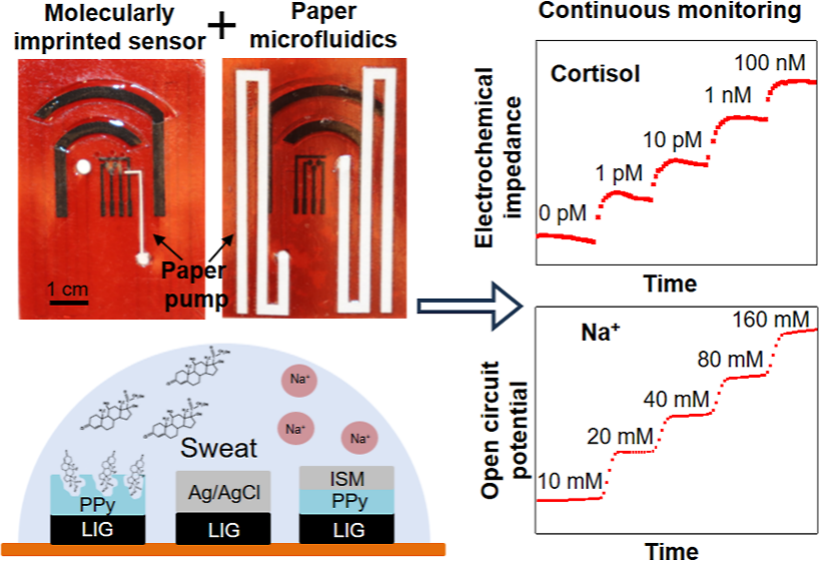

The urgent need for real-time and noninvasive monitoring of health-associated biochemical parameters has motivated the development of wearable sweat sensors. Existing electrochemical sensors show promise in real-time analysis of various chemical biomarkers. These sensors often rely on labels and redox probes to generate and amplify the signals for the detection and quantification of analytes with limited sensitivity. In this study, we introduce a molecularly imprinted polymer (MIP)-based biochemical sensor to quantify a molecular biomarker in sweat using electrochemical impedance spectroscopy, which eliminates the need for labels or redox probes. The molecularly imprinted biosensor can achieve sensitive and specific detection of cortisol at concentrations as low as 1 pM, 1000-fold lower than previously reported MIP cortisol sensors. We integrated multimodal electrochemical sensors with an iontophoresis sweat extraction module and paper microfluidics for real-time sweat analysis. Several parameters can be simultaneously quantified, including sweat volume, secretion rate, sodium ion, and cortisol concentration. Paper microfluidic modules not only quantify sweat volume and secretion rate but also facilitate continuous sweat analysis without user intervention. While we focus on cortisol sensing as a proof-of-concept, the molecularly imprinted wearable sensors can be extended to real-time detection of other biochemicals, such as protein biomarkers and therapeutic drugs.

## Introduction

Rapid and noninvasive quantification of biochemical parameters associated with individual health and disease conditions can facilitate timely clinical intervention and personalized medicine.^1−4^ This unmet need has motivated research efforts in developing wearable sweat sensors to enable real-time analysis of various chemical biomarkers, including ions, metabolites, hormones, and proteins.^5−9^ For example, wearable sweat sensors have been developed to monitor psychological and physiological stress by measuring the concentration of cortisol, a stress biomarker. These sensors rely on biorecognition elements, including antibodies,^10,11^ aptamers,^12−14^ and molecularly imprinted polymers (MIPs),^12,15,16^ to achieve specific and sensitive cortisol quantification. Antibody-based sensors typically involve add-on reagents, nanoparticle labeling, and enzymatic reaction, making in situ, real-time quantification challenging. In addition, antibodies are less stable than synthetic biorecognition elements, such as aptamers and MIPs.^17−19^ Several aptamer-based electrochemical sensors show promise in monitoring molecular targets in real time, although the development process of aptamers can take several months and is cost-intensive.^20−22^ MIPs rely on binding pockets with complementary shapes and chemical functionality to molecular targets to provide affinity and specificity, which are cost-effective, easy to synthesize, and generalizable to a wide range of molecular targets.^23−25^ Although MIP-based electrochemical sensors are low-cost and can offer real-time molecular detection, it is challenging to achieve molecular detection at a picomolar concentration. A molecularly imprinted wearable sensor that is label and redox probe-free and highly sensitive and selective would expand the capability of detecting and quantifying low-abundant molecular biomarkers.

Recent advances in integrating microfluidics with sensors allow simultaneous quantification of sweat loss, sweat rate, and biochemicals, which can decipher the intrinsic correlation between sweat rate and biochemical concentration in sweat.^26−28^ For example, a class of thin, silicone microfluidic devices with embedded colorimetric sensors can capture and handle sweat and measure total sweat loss, sweat rate, and various chemical parameters.^29−31^ Control over the sweat flow in the microfluidic device can be achieved by varying microchannel dimensions and internal structures such as capillary valves and superabsorbent polymer valves.^30,31^ The sophisticated design of pinch valves and suction pumps can purge sweat to reset a device to an empty state through manual operation.^32,33^ Various flexible materials, including polydimethylsiloxane, polyethylene terephthalate, adhesives, and paper, have been utilized to construct wearable microfluidic devices for sweat analysis.^28^ These hydrophobic polymeric materials can be modified to improve surface-wetting properties and reduce flow impedance for sweat transportation and sweat rate quantification.^32,34^ Alternatively, paper microfluidics-enabled plasmonic sensors have been designed to quantify sweat volume, rate, and sweat biomarkers.^35^ Paper microfluidics offer advantages, including low cost, high absorbency, rapid flow transportation through capillary action, and easy integration of functional materials for biosensing.^36−40^ The ability to integrate paper microfluidic channels and pumps with flexible electronic devices would enable low-cost lab-on-skin systems and operation without user intervention.

Here, we report a flexible wearable device that can induce sweat via iontophoresis on demand and simultaneously quantify several parameters, including sweat volume, secretion rate, sodium ion, and cortisol concentrations. The integrated paper microfluidic module enables the quantification of sweat volume and secretion rate and also function as a pump to remove the collected sweat from the sensing chamber once the chamber is filled. This reset approach does not involve user intervention and eliminates the mixing of sweat collected at different times. The MIP was electrochemically synthesized on laser-induced graphene (LIG) electrodes, which enables cortisol binding to occur within the Debye length for high sensitivity. The MIP-based sensor detects and quantifies the cortisol level in sweat without the presence of labels and redox probes using electrochemical impedance spectroscopy. The ionic selective membrane-coated electrode was used to quantify sodium ion concentration through open circuit potential (OCP) measurements. We demonstrated that the wearable device can be used to measure these sweat parameters on a healthy human subject.

## Results and Discussion

### Design of Wearable Sensor with Paper Microfluidics

**Figure 1:**
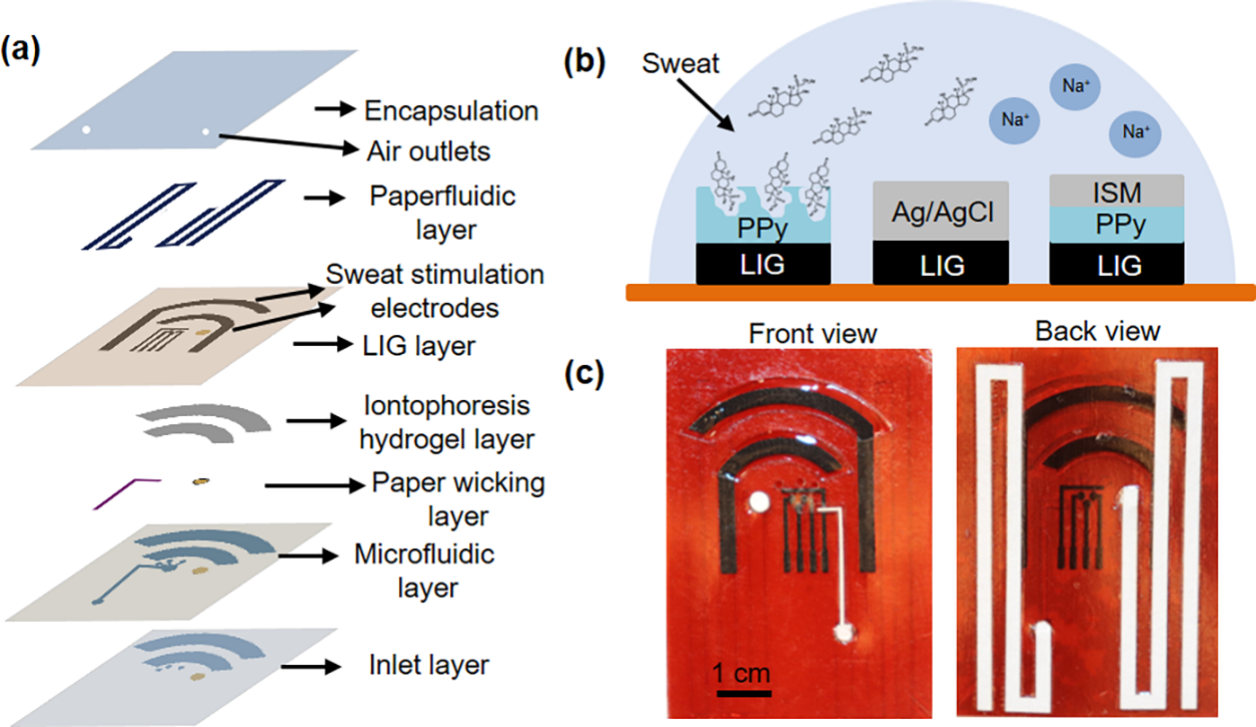
Wearable device design. (a) Stacked view of the wearable device showing various functional layers and components. (b) Schematic illustration of the electrochemical sensor design, including a LIG counter electrode (not shown), an Ag/AgCl-coated LIG reference electrode, a cortisol-specific MIP LIG electrode, and a sodium ion-selective membrane (ISM) coated polypyrrole (PPy)/LIG electrode. (c) Optical images of the assembled device. The front view image shows the skin-interfaced side.

The wearable device is composed of several functional modules, including an iontophoresis sweat induction module, multimodal biosensors, and microfluidic modules (Figure 1a). The iontophoresis sweat induction module comprises two arc-shaped graphene electrodes coated with hydrogels to stimulate sweat secretion on demand. The anode electrode coated with a carbachol-loaded hydrogel layer is close to the microfluidic inlet where the secreted sweat is captured. The cathode electrode coated with sodium chloride hydrogel completes the electrical circuit. The microfluidic module transports sweat from the inlet to the sensing chamber and then to the paper microfluidic (simplified as paperfluidic) layer. The sensing chamber and inlet are defined by a double-sided skin adhesive, forming a mechanically robust interface between the skin and the wearable device. The inlet has three circular openings with a 1 mm diameter to collect sweat and transport it to the sensors for quantifying sodium ion (Na^+^) and cortisol concentrations. One paper strip (0.5 mm wide and 2.2 cm long) serves as a pump and connects the outlet of the sensing chamber to the paperfluidic layer on the backside of the device to efficiently remove the accumulated sweat. Another inlet has one circular opening with a 2 mm diameter connected to the paperfluidic layer for sweat volume and rate quantification. Several via holes in these layers were filled with wicking paper to facilitate sweat transportation. Finally, the device was encapsulated with a thin Tegaderm transparent film to minimize sweat evaporation and protect it from environmental contamination. The encapsulation film has two outlets near the end of the paperfluidic layer to eliminate back pressure that can impede sweat flow.

We employed laser scribing to fabricate porous LIG electrodes on a flexible polyimide substrate, including sweat induction and sensing electrodes. This fabrication approach is simple, inexpensive, scalable, and enables mask-free graphene patterning on various substrates.^41−43^ The electrochemical sensors contain four electrodes, including one LIG electrode as a counter electrode, one LIG coated with Ag/AgCl as a reference electrode, and two working electrodes (Figures 1b and S1). One working electrode is LIG with electrochemically synthesized cortisol-specific MIP for cortisol detection and quantification. The other working electrode is LIG coated with PPy and sodium ion-selective membrane to quantify sweat Na^+^ concentration. Figure 1c shows the optical images of the device with all layers assembled. The front view image shows the side interfaced with the skin. The two arc-shaped openings are coated with hydrogel layers, which are directly in contact with the skin, for sweat stimulation. The front view also shows a circular opening connected to the paperfluidic and three small inlets for capturing and transporting the sweat to the sensing chamber. The back view image highlights two paperfluidic channels, with the left channel removing the accumulated sweat from the sensing chamber at a well-defined rate and the right channel quantifying sweat volume and secretion rate. We chose chromatography paper to construct paperfluidic channels because it has a well-defined flow rate and absorption capacity.

### Microfluidic Characterization and Sweat Volume/Rate Quantification

**Figure 2:**
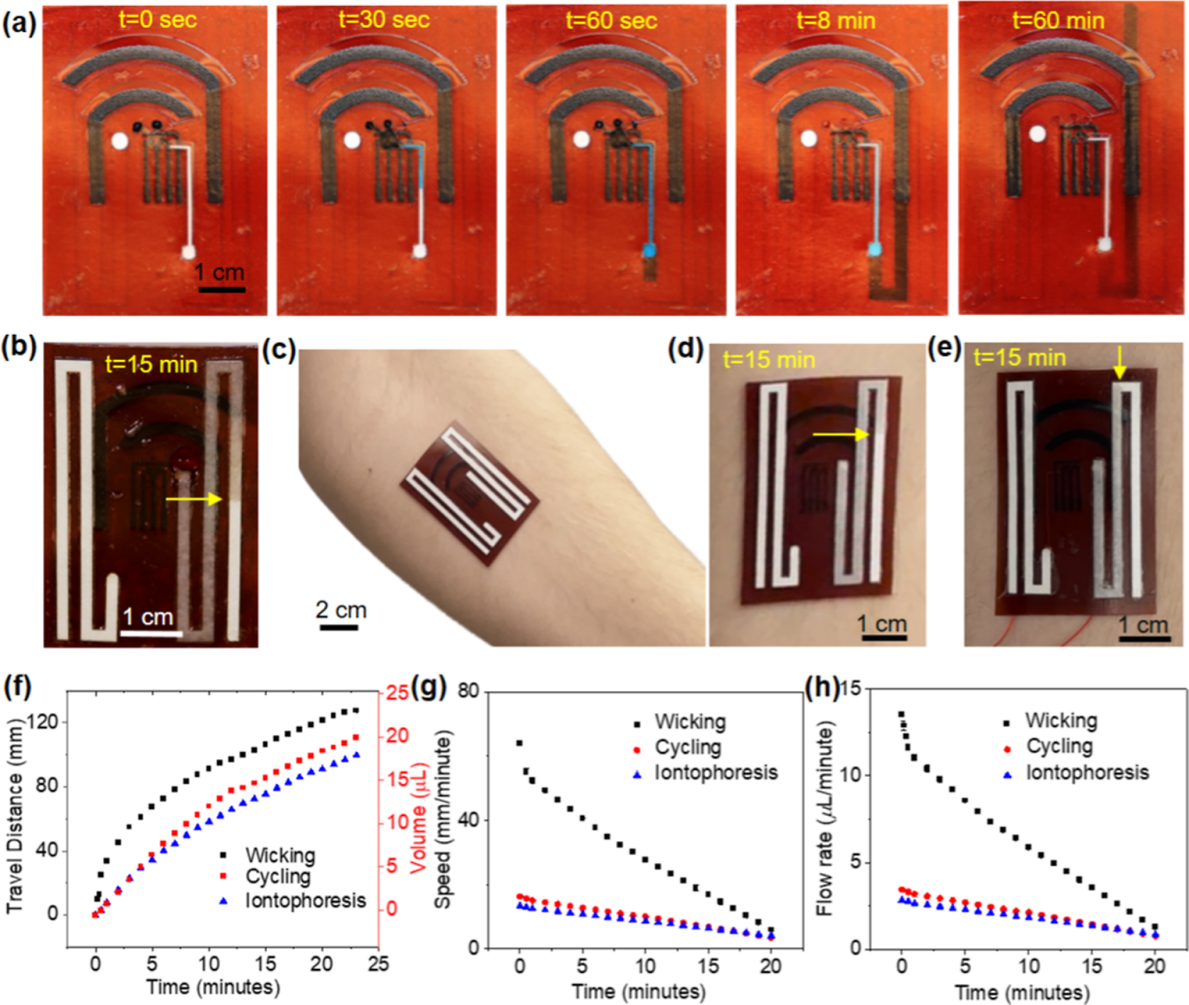
Characterization of paper microfluidics. (a) Optical images of a device collected at different times after adding an aqueous blue dye solution and then water to the inlets of the sensing chamber. (b) Optical image of a device collected at 15 min after introducing fluid to the inlet of a paper microfluidic channel. The arrow points to the fluid front. (c) Optical image of a device on the forearm of a human subject showing sweat collection after 15 min of (d) cycling and (e) iontophoresis. (f) Comparison of liquid travel distance and volume as a function of time for wicking, iontophoresis, and cycling. (g) Comparison of paper wicking speed and iontophoresis and cycling-induced sweat travel speed along the paperfluidic channel. (h) Comparison of paper wicking rate and sweat secretion rate induced by iontophoresis and cycling.

The wetting properties of microfluidic device surfaces have a significant impact on fluid flow characteristics. We treated the LIG on the polyimide substrate with oxygen plasma to enhance the hydrophilicity of surfaces. The untreated surface had a contact angle of 109.3 ± 3.8°, while the freshly plasma-treated and one-year-old treated surfaces showed contact angles of 6.5 ± 0.3° and 7.5 ± 0.4°, respectively (Figure S2). These results demonstrate that plasma treatment makes the LIG surface hydrophilic, and this hydrophilicity is maintained even after one year. Figure 2a demonstrates that an aqueous blue dye solution introduced to the inlet of the device can rapidly fill the sensing chamber in less than 30 s and be removed by the paperfluidic pump effectively over time. After 60 s, we introduced additional water to the inlet and observed the dye solution was completely removed in 8 min. The paperfluidic pump can continuously transport the dye solution through capillary force and remove accumulated fluid from the sensing chamber without any sign of backflow. This prevents the mixing of sweat samples generated sequentially, which facilitates the accurate quantification of analytes with varying concentrations. Without oxygen plasma treatment, the dye solution remained at the inlet and could not flow into the sensing chamber due to the poor wetting properties of untreated LIG and polyimide (Figure S3).

We characterized the liquid-wicking kinetics of paper microfluidics assembled on the polyimide and encapsulated by a thin Tegaderm transparent film and evaluated their capability to quantify the sweat volume and rate in real time. The fluid flow rate in the paperfluidic channel was quantified by video recordings of fluid propagation after introducing an excess amount of fluid to the inlet. It is easy to visualize the fluid front on the paper substrate (Figures 2b and S4). The fluid traveled 106 mm along a 2 mm wide paperfluidic channel in 15 min. The fluid flow follows the Lucas-Washburn equation, which quantifies the correlation between travel distance, surface tension, viscosity of the liquid, and contact angle between the fluid and boundary wall and time.^44^ Although the paper wicking rate slows down over time, it remains higher than the typical human sweat rate of 12–120 μL/cm^2^·h.^45^ For comparison, we quantified the sweat rate induced by intense exercise and iontophoresis with the paperfluidic devices applied on the forearm of a healthy human subject (Figures 2c, S5 and S6). Figure 2d shows the optical image of the device collected at 15 min following intense cycling exercise, which showed the sweat travel distance of 86 mm. Figure 2e shows the optical image of the device collected at 15 min following iontophoresis, which involves a small current of 100 μA applied through the sweat stimulation electrodes for 5 min. Compared to intense exercise, iontophoresis-induced sweat showed a shorter travel distance, suggesting a lower sweat volume (Figure 2e). Figure 2f shows the comparison between the fluid travel distance upon wicking and sweat travel distance with iontophoresis and exercise over time. The travel distance linearly increases with increasing volume. The volume in Figure 2f is derived from our previous work,^35^ which used a controlled quantity of liquid to establish the relationship between the travel distance and volume of liquid uptaken by the paper microfluidic channel. The liquid volume and travel distance relationship is 4.74 ± 0.16 mm/μL for the paper with a channel width of 2 mm.^35^ The sweat rates in both cases slowed down over time, but they were much slower than the paper wicking rate (Figure 2g,h). These results confirm the capability of the paperfluidic module for real-time sweat volume and rate quantification.

### LIG Electrode Characterization

**Figure 3:**
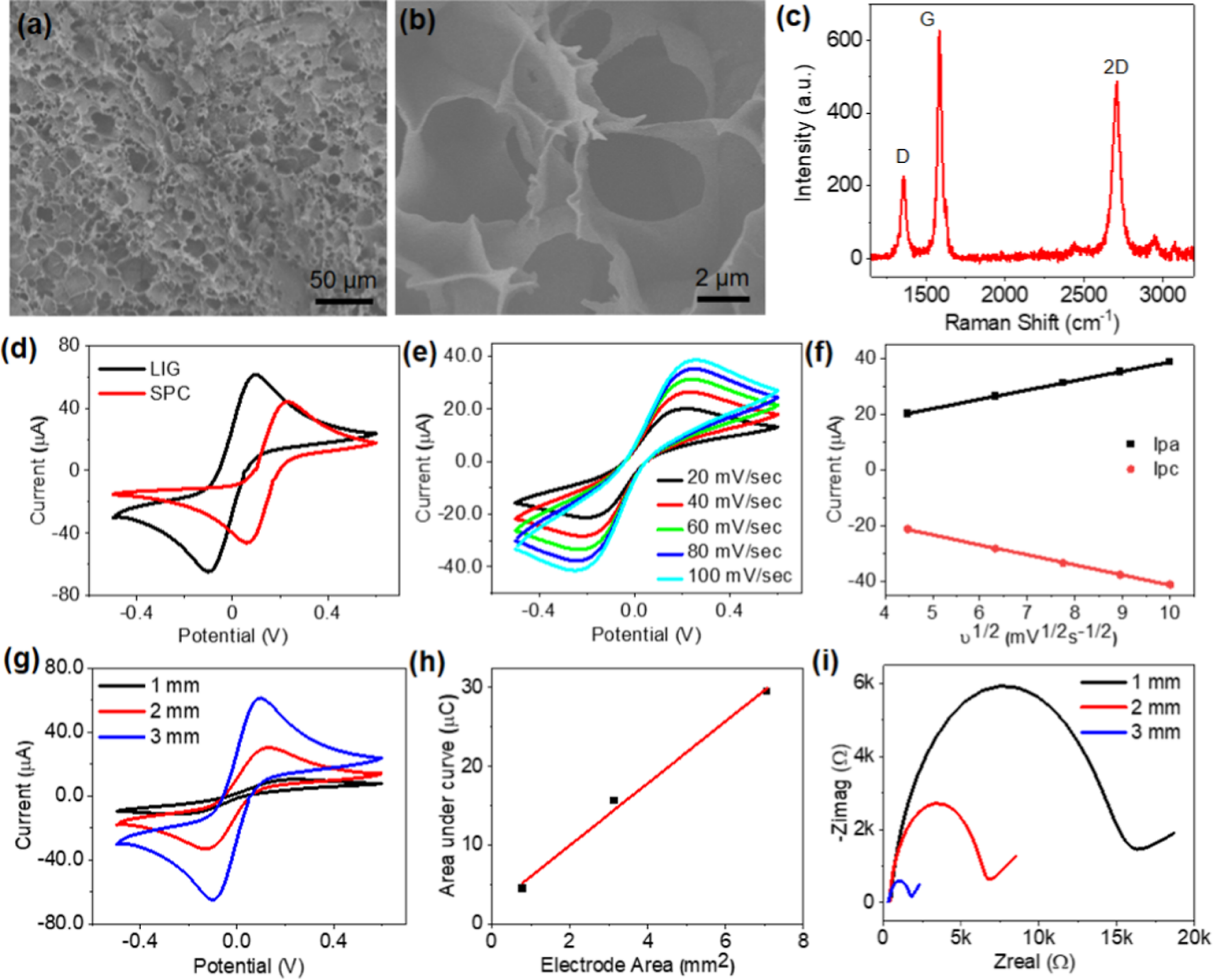
LIG electrode characterization. (a) Low-magnification and (b) high-magnification SEM images of LIG. (c) Raman spectrum of LIG. (d) Comparison of cyclic voltammograms collected with a commercial screen-printed carbon (SPC) electrode and LIG electrode of 3 mm in diameter. (e) Cyclic voltammograms collected with the LIG electrode of 2 mm in diameter at changing scan rates and (f) corresponding anodic and cathodic currents as a function of the square root of scan rates. (g) Cyclic voltammograms collected with the LIG electrodes of varying diameters and (h) corresponding area under the curve with respect to electrode area. (i) Nyquist plots for the LIG electrodes of varying diameters.

With the laser scribing approach, it is easy to fabricate LIG electrodes and interconnects of different dimensions and geometries (Figure S7). The scanning electron microscope (SEM) images reveal a highly porous structure of graphene induced by laser scribing (Figure 3a,b). We collected LIG Raman spectra with a Raman spectrometer at 514 nm, which show three prominent peaks, including *D*, *G*, and 2*D* Raman peaks at ∼1350, 1580, and 2700 cm^–1^, respectively (Figure 3c). The *D* peak originates from the defect active breathing modes of six-atom rings.^46^ The G and 2*D* peaks correspond to the high-frequency E_2g_ phonon and the second-order zone-boundary phonons, respectively.^47^ The 2*D* peak can be fitted with a single Lorentzian peak centered at ∼2700 cm^–1^, indicating the LIG is primarily comprised of single-layer graphene (Figure S8).^47^

We compared the electrochemical performance of a 3 mm-diameter LIG electrode with a commercial SPC electrode of the same dimension. Figure 3d shows the cyclic voltammograms collected with two electrodes in K_4_Fe(CN)_6_/K_3_Fe(CN)_6_ as a reference system. The oxidation–reduction signature peaks of ferro-ferricyanide show that the LIG electrode has a higher current response than the SPC electrode. This can be attributed to the graphene’s high surface area and electron mobility. Electrodes with changing diameters of 1, 2, and 3 mm can be reliably fabricated to test their electrochemical performance and sensitivity for cortisol quantification (Figure S7). The cyclic voltammograms collected with the LIG electrode of 2 mm in diameter show that the current increases with increasing scan rates from 20 to 100 mV/s (Figure 3e). The peak anodic current (*I*_pa_) and peak cathodic current (*I*_pc_) follow a linear relationship with the square root of the scan rate, suggesting a diffusion-limited voltammetry response (Figure 3f).^48^

Cyclic voltammograms were also collected with LIG electrodes of varying diameters to estimate the effect of miniaturizing the electrode on the electrochemical performance (Figure 3g). The cyclic voltammetric response shows that the current increases with increasing electrode size (Figure 3g). The maximum current is obtained for a 3 mm working electrode. The charge, determined by integrating the current with respect to potential, linearly increases with increasing the electrode area (Figure 3h). This confirms that the electrodes of varying diameters fabricated with the laser scribing approach provide a consistent electrochemical response. The Nyquist plots show that both real and imaginary magnitudes of impedance increased with decreasing electrode diameters (Figure 3i). Figure S9 shows the electrochemical impedance magnitude and phase angle of the electrodes recorded at the frequency range from 0.1 Hz to 100 kHz. The increased impedance with decreasing electrode size is consistent with previous reports.^49,50^ We employed the LIG electrodes with 1 mm diameter to construct sodium ion and cortisol sensors described below, as it only takes ∼2.4 μL sweat to fill the miniaturized sensing chamber.

### MIP Synthesis and Characterization

**Figure 4:**
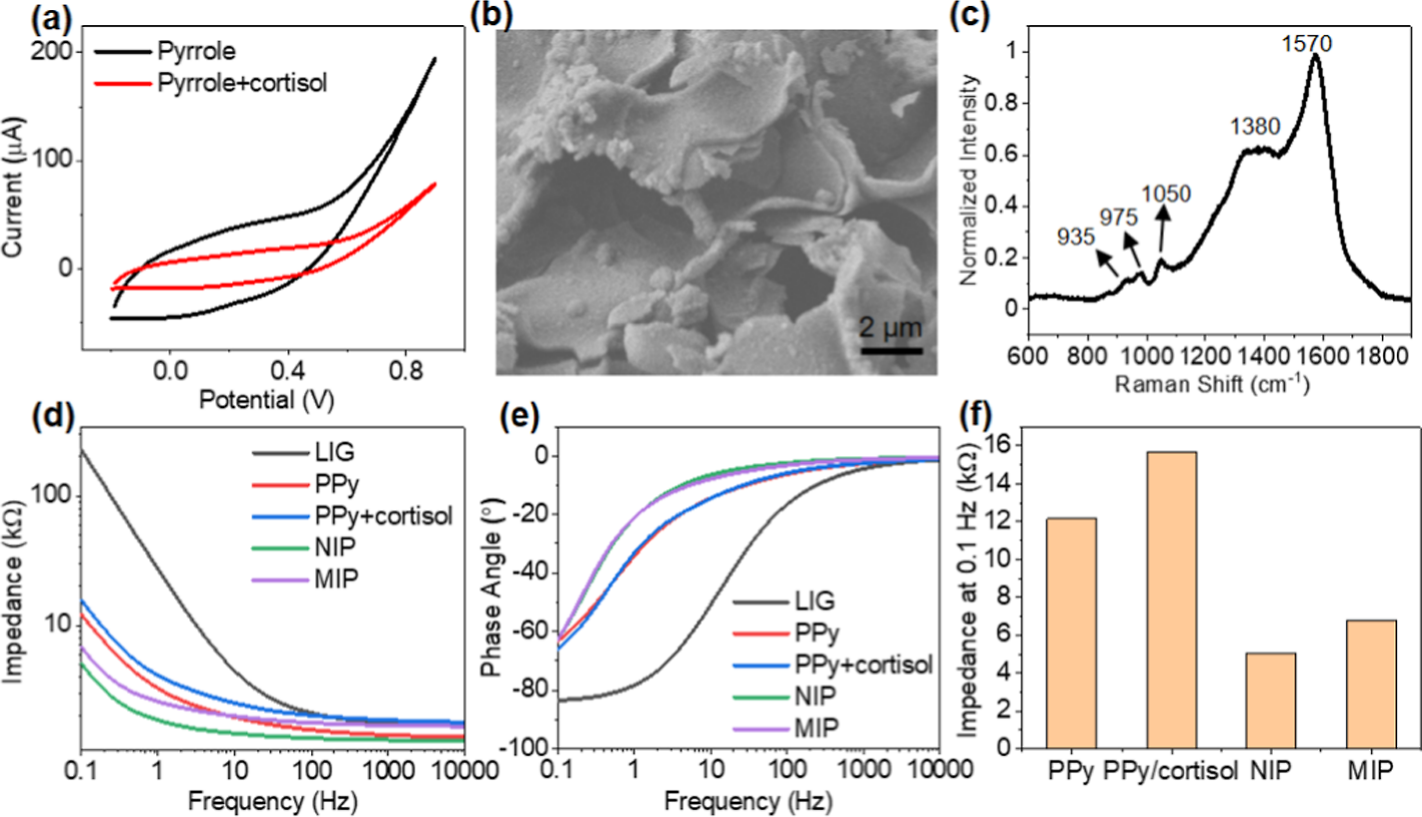
MIP functionalization of LIG electrode. (a) Cyclic voltammetric curves corresponding to the 10th electrodeposition cycle of PPy with and without cortisol. (b) High-magnification SEM image of PPy-coated LIG. (c) Raman spectrum of the PPy-coated LIG. (d) Impedance magnitude and (e) phase angle of LIG, PPy-coated LIG with and without cortisol, MIP, and NIP LIG electrodes. (f) Impedance magnitude comparison of PPy-coated LIG, MIP, and NIP LIG electrodes at 0.1 Hz.

We employed pyrrole as a monomer to synthesize MIP on LIG via electrochemical deposition in the presence of cortisol templates. After PPy deposition, the cortisol templates were removed from PPy to yield MIP for cortisol detection and quantification. The PPy without cortisol, i.e., nonimprinted polymer (NIP), serves as a control in evaluating the sensing performance of MIP-based cortisol sensors. Figure 4a shows the cyclic voltammetry curves corresponding to the 10th electrodeposition cycle of PPy using pyrrole in phosphate-buffered saline (PBS) with and without cortisol. The current was higher during the electrodeposition of the NIP than the MIP due to the presence of nonconducting cortisol inside the MIP matrix. SEM image reveals a thin layer of ∼100 nm PPy uniformly deposited on the graphene flakes (Figure 4b). Representative Raman spectrum collected from PPy-coated LIG shows prominent Raman peaks at ∼1380 and 1570 cm^–1^, which are convoluted with *D* and *G* Raman peaks from graphene (Figure 4c). The peaks between 1380 and 1570 cm^–1^ are attributed to the backbone C=C bonds stretching, C–N stretching, and inter-ring stretching C–C vibration mode of PPy.^51,52^ The asymmetric band at ∼1050 cm^–1^ is attributed to C–H stretching vibration bands of polarons and bipolarons.^53^ The 975 and 935 cm^–1^ peaks correspond to ring deformation associated with the polaron and bipolaron bands, indicating the oxidized form of PPy.^51^

Following each step of the LIG electrode modification, we recorded the electrode impedance in PBS at frequencies ranging from 0.1 Hz to 10 kHz (Figure 4d–f). The results show that the PPy coatings significantly decreased the LIG electrode impedance magnitudes and phase angles due to the high conductivity of the oxidized PPy in the low-frequency range (Figure 4d). At 0.1 Hz, the impedance of the PPy-coated electrode is 10-fold lower than the LIG electrode. The electrode with cortisol-embedded PPy exhibited higher impedance than the PPy without cortisol because cortisol hindered the flow of electrons and ions between the electrode and the surrounding electrolyte. After exposing the PPy-coated LIG electrodes to a mixture of acetic acid and methanol (7:3 v/v) to remove the cortisol templates, the electrochemical impedance further decreased due to the enhanced electrical conductivity of LIG electrodes following the chemical treatment with acetic acid.^54^ The impedance of NIP and MIP electrodes at 0.1 Hz decreased by half compared to that before exposing the electrodes to the template removal solution (Figure 4f). The resultant MIP electrode showed a slightly higher impedance than the NIP. The higher impedance of the MIP electrode compared to the NIP electrode may result from two factors: the inherently higher resistance of the MIP electrode due to less deposition of PPy in the presence of cortisol and the residual cortisol templates left inside the MIP after the removal process. Considering wearable applications, we measured the electrochemical impedance and electrical resistance of PPy-coated LIG electrodes in a flat position and under bending (Figure S10). The impedance and resistance remain stable upon bending at a small radius of 2.4 cm, confirming that the devices can be applied to the forearm of pediatric and adult individuals and provide consistent measurements.

### MIP Cortisol Sensor Performance

**Figure 5:**
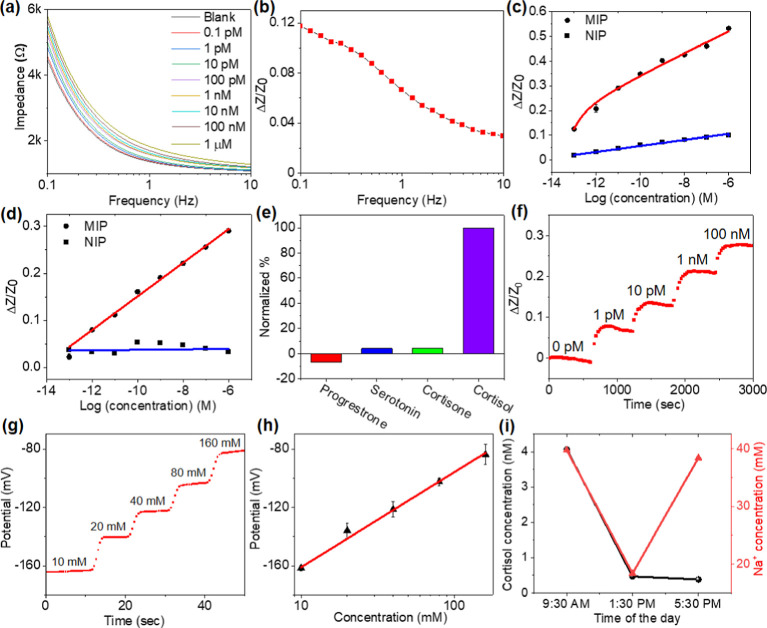
MIP-electrochemical sensor performance. (a) Impedimetric response of a MIP sensor to varying concentrations of cortisol from 0.1 pM to 1 μM. (b) Normalized change in impedance at 0.1 pM cortisol as a function of frequency. (c) Normalized impedance changes with respect to cortisol concentrations for MIP and NIP Electrodes (c) without and (d) with oxygen plasma treatment. (e) Comparison of impedance changes after exposing the MIP sensor to interfering molecules and cortisol. (f) Real-time impedance changes of the MIP sensor after exposing it to changing concentrations of cortisol. (g) Real-time OCP of the ISM-coated electrodes after exposure to varying Na^+^ concentrations. (h) Calibration curve of OCP as a function of Na^+^ concentrations. (i) Sweat cortisol and Na^+^ concentrations quantified at different times of the day. Error bars in (c,d,h) represent the standard deviation of the measured values (*n* = 3).

Next, we investigated the sensitivity and specificity of MIP cortisol sensors and demonstrated the capability of monitoring cortisol in real time. A MIP cortisol sensor comprises three LIG electrodes, including one counter electrode, one Ag/AgCl-coated LIG as a reference electrode, and one MIP-LIG electrode as a working electrode. The devices with an NIP-LIG working electrode were also prepared for comparison. Using these three electrode devices, we mainly analyzed the impedance changes of MIP-LIG/NIP-LIG electrodes upon exposure to cortisol at the low-frequency range. We exposed a MIP sensor to varying concentrations of cortisol from 0.1 pM to 1 μM in pH 6.5 artificial sweat and recorded the electrochemical impedance at the frequency range from 0.1 to 10 Hz (Figure 5a). The impedance increased with increasing concentrations of cortisol, and the blank condition represents the artificial sweat without cortisol. The impedance increase induced by the captured cortisol at 0.1 pM concentration normalized with respect to the blank impedance (Δ*Z*/*Z*_0_) decreased with increasing frequency, following the same trend as the absolute impedance (Figure 5b). The maximal impedance change (Δ*Z*/*Z*_0_) was obtained at 0.1 Hz, which was used for the following analysis. We compared the dose–response curves collected from MIP and NIP devices after the devices were exposed to the cortisol solution (Figure 5c). The MIP device exhibited an increase in impedance with increasing cortisol concentrations, while the NIP device showed negligible changes. The MIP device can detect cortisol at concentrations from 0.1 pM to 1 μM. The plasma treatment was applied to modify the microfluidic surface to become hydrophilic, thereby facilitating sweat transport to the sensing chamber. However, this treatment may also alter the interaction between cortisol and the MIP. To assess the effect of plasma treatment on sensitivity, we conducted impedance measurements using the oxygen plasma-treated devices (Figure 5d). After the plasma treatment, the overall impedance changes measured by the MIP device decreased while the linearity of impedance changes as a function of cortisol concentrations improved. The impedance changes in the NIP device remained negligible. The plasma-treated MIP device can detect and quantify cortisol at a low concentration of 1 pM, 1000-fold lower than previously reported MIP-based electrochemical sensors.^12,15,16^ These results confirm that the MIP electrochemical sensor can provide highly sensitive cortisol quantification with electrochemical impedance measurements. The strong binding of cortisol to the MIP cavity is primarily due to a combination of hydrogen bonding and hydrophobic interactions. Although cortisol is uncharged, its binding alters the ion distribution on the charged PPy surface within the Debye length, resulting in impedance changes that enable the highly sensitive detection of cortisol. In these conductive MIP-based electrochemical sensors, the binding events occur within the Debye length, which is less than 1 nm under physiological conditions. This overcomes the charge screening limitations in electrochemical sensors that rely on nonconductive biorecognition elements.^55^

To validate the selectivity of the MIP cortisol sensor, we exposed the sensor to several interfering molecules at physiologically relevant concentrations, including progesterone (200 pM), serotonin (10 nM), and cortisone (25 nM) (Figure 5e). Among these, cortisone and cortisol have similar chemical structures. The impedance changes from the interfering molecules remain below 7% of changes from 10 nM cortisol, confirming the high selectivity of the MIP cortisol sensor. To evaluate the real-time cortisol quantification, we constructed the MIP sensor in a microfluidic device and introduced the changing concentrations of cortisol from 0 pM to 100 nM in sequential (Figure 5f). The electrochemical impedance was continuously recorded with a time interval of 27 s. We observed a rapid increase in the impedance in the first 2 min after introducing a different concentration of cortisol, and the impedance reached a plateau in ∼3 min. The impedance changes are consistent with those in the calibration curve shown in Figure 5d. These results demonstrate that the MIP sensor can continuously quantify the cortisol concentration in real time. It is important to note that the channel width of the paperfluidic pump can be optimized to control the sweat removal rate based on the sensor’s equilibrium time. It takes ∼2 min to fill the sensing chamber of ∼2.4 μL if sweat is rapidly produced with iontophoresis and intense cycling (Figure 2f). The complete removal of the sweat with the paperfluid pump with a 0.5 mm wide channel width takes 3 min, allowing the sensor to reach equilibrium. With this design, the sensor accuracy is not affected by varying sweat secretion rates. The continuous quantification here is limited to increasing concentrations of cortisol because refreshing the MIP surface in situ is challenging. Despite this limitation, the capability of monitoring the increasing cortisol levels could still provide valuable insights into periods of heightened stress, allowing individuals to identify triggers and implement stress-reduction techniques.

### Sweat Sodium Ion Concentration Quantification

Sweat sodium ion concentration can reflect electrolyte imbalance-associated medical conditions, such as hyponatremia, muscle cramps, and dehydration.^56−58^ The physiological concentration of sodium ions in sweat falls in the range between 10 and 90 mM.^59^ We incorporated Na^+^ selective membrane on top of the PPy-modified LIG electrode to quantify the sodium ion concentration. The OCP of the electrode increased after exposing it to increasing Na^+^ concentrations from 10 to 160 mM (Figure 5g). The sensitivity was calculated to be around 64.9 mV/decade (Figure 5h), which is close to the theoretical value of 59 mV/decade according to the Nernstian equation. We confirmed the pH stability of the Na^+^ ion sensor by exposing it to artificial sweat with varying pH levels, ranging from pH 5 to pH 7 (Figure S11). The pH of the artificial sweat was adjusted with hydrochloric acid and potassium hydroxide. These results also demonstrate the consistent response of the Na^+^ sensor after exposure to varying concentrations of common ions in sweat, including potassium, hydrogen, chloride, and hydroxide.

We validated the wearable device’s performance for quantifying the Na^+^ and cortisol concentrations in situ on a healthy subject using a freshly prepared device each time. The subject cycled on a stationary bike for 10 min at different times of the day (9:30 a.m., 1:30 p.m., and 5:30 p.m.) to produce sweat. The average sweat rates were quantified to be ∼2 μL/min. The sweat Na^+^ concentration varied between 19 and 40 mM (Figure 5i). This falls into the normal range for the sweat produced by healthy individuals.^59^ Many factors can contribute to the fluctuation in ion concentration, such as dietary and water intake and physical load. Figure 5i shows the cortisol levels at different times of the day. The cortisol level in the morning was quantified to be 4 nM, ten times higher than that in the afternoon. The decreasing trend follows the human circadian rhythm of cortisol expression, in which cortisol levels rise at the beginning of the biological day and decline throughout the day.^60^

## Conclusions

In summary, the MIP-based electrochemical impedance biosensor reported here does not rely on labels or redox probes to achieve sensitive and specific detection and quantification of molecular biomarkers in sweat. The wearable sweat sensor combined with a sweat induction module and paper microfluidics enables real-time, simultaneous quantification of multiple parameters, including sweat volume, rate, sodium ion, and cortisol concentrations. We demonstrate that the MIP-functionalized LIG sensor can provide real-time cortisol quantification at a low concentration of 1 pM, 1000-fold lower than previously reported MIP-based electrochemical sensors. The ion-selective membrane functionalized LIG electrodes can be used to quantify sweat sodium concentration at physiologically relevant ranges via OCP measurements. The paper microfluidics can quantify the sweat volume and secretion rate and also reset the sensing chamber to continuously analyze the generated sweat. Although the current study focuses on cortisol sensing as a proof-of-concept, the MIP-based electrochemical sensors can extend to real-time detection and quantification of other biochemicals of interest, such as protein biomarkers and therapeutic drugs. The simultaneous multiparameter quantification approach could facilitate the diagnosis and monitoring of medical conditions based on the concentration of these biochemical biomarkers.

## Experimental Section

### Materials

Pyrrole, cortisol, cortisone, serotonin, progesterone, potassium ferrocyanide (II), potassium ferricyanide (III), silver nitrate, iron chloride, sodium ionophore, sodium tetrakis[3,5-bis(trifluoromethyl)phenyl]borate, polyvinyl chloride and bis(2-ethylhexyl) sebacate was purchased from Sigma-Aldrich. 10× PBS was purchased from Gibco. Carbachol, cellulose chromatography paper (Whatman #1 grade), polyvinyl butyral (PVB), sodium citrate dihydrate, sodium chloride, iron(III) chloride, potassium hydroxide, Acetic acid, methanol, and sodium hydroxide were purchased from Fisher Scientific. Artificial eccrine sweat (pH 4.5) was obtained from Biochemazone. The pH of the artificial sweat was adjusted to pH 5–7 using an aqueous solution of 10 mM potassium hydroxide. The SPC electrode was procured from Zensor. Polyimide films of 150 μm thick were obtained from CS Hyde Company. Medical grade double-sided adhesive (2477P) was purchased from 3M. Type 1 deionized water (18.2 mΩ·cm) was produced by the Sartorius Arium Pro Ultrapure water system. The LIG electrodes were plasma treated with high intensity for 1 min using a plasma cleaner PDC-001 from Harrick Plasma.

### Fabrication and Modification of the LIG Electrodes

A 70 W CO_2_ laser cutter (LS1630, Boss Laser) was used to fabricate LIG electrodes. The optimized fabrication parameters for electrodes and interconnects are power 10% and speed 55 mm/s. Electrode with varying diameters of 1, 2, and 3 mm were fabricated. The flexible wires were bonded to the devices using heat-curable silver ink (Creative Materials). The interconnects were encapsulated with Tegaderm transparent film (3M). Ag/AgCl reference electrodes were prepared by the electrodeposition of silver on LIG electrodes using a mixture of silver nitrate and sodium citrate dihydrate as precursors with cyclic voltammetry from −0.9 to 0.9 V with 100 mV/s scan rate using Reference 620+ Potentiostat (Gamry Instruments). After the electrodeposition, silver-coated LIG electrodes were exposed to 0.1 M FeCl_3_ aqueous solution for 30 s to obtain Ag/AgCl reference electrodes. To stabilize the reference electrodes, a cocktail of 79.1 mg of PVB and 50 mg of sodium chloride (NaCl) in 1 mL of methanol was added to the electrode surface and dried overnight.

The cortisol MIP was synthesized on the LIG using the electropolymerization method. The LIG electrodes were exposed to a precursor solution of 37.5 mM pyrrole and 5 mM cortisol in 1X PBS and subjected to 10 cyclic voltammetry cycles from −0.2 to 0.9 V with a scan rate of 50 mV/s. The electrodes were immersed in a solution of acetic acid and methanol (7:3 v/v) for 30 min to extract cortisol templates, followed by thorough rinsing with deionized water. The NIP was synthesized using the same procedure as MIP, except that no cortisol was present in the polymerization solution.

The Na^+^ selective membrane was fabricated on a PPy-coated LIG electrode. The PPy-coated LIG electrode was prepared the same way as the MIP electrode except cortisol was not added to the precursor solution. A cocktail was first prepared by dissolving 2 mg of Na ionophore X, 1.1 mg sodium tetrakis[3,5 bis(trifluoromethyl)phenyl]borate, 66 mg polyvinyl chloride, and 130.9 mg bis(2-ethylhexyl) sebacate into 1320 μL of tetrahydrofuran. This cocktail was then drop-casted on the PPy-coated LIG electrode, followed by overnight drying. The electrode was subsequently conditioned in an aqueous solution of 100 mM sodium chloride overnight.

### Microfluidic Device Characterization

The functional layers highlighted in Figure 1a were assembled to form microfluidic devices. A blue dye aqueous solution was added to the inlets, and the flow profile of the microfluidic device was captured by video recordings. The wet properties of polyimide films with LIG electrodes with and without plasma treatment were compared. After the dye solution filled the sensing chamber, water was introduced to the inlets to evaluate the refreshability of the sensing chamber using the paperfluidic pump. The wicking rate of the paper microfluidics was quantified by video recording the fluid propagation after adding excess water to the inlet of the paper microfluidic channel.

The sweat secretion rate and volume were calculated from the video recordings of the paperfluidic devices. A healthy human subject of 22 years old performed 30 min of cycling while wearing the paperfluidic device on the forearm. For sweat stimulation, anode hydrogels were prepared by mixing 2 wt % carbachol and 2.4 wt % agarose solution at 100 °C and then added to the LIG electrode surface, followed by cooling at room temperature for solidification. Cathode hydrogels were prepared by replacing carbachol with 1 wt % sodium chloride. After applying the device on the forearm of the human subject, a small current of 100 μA was applied through the sweat stimulation electrodes to the skin for 5 min to induce sweat. Following the stimulation, the sweat secretion rate was quantified from the video recordings.

### Material and Sensor Characterization

SEM images of LIG and PPy-coated LIG were collected with field effect-scanning electron microscopy (JEOL JSM-7500F). The Raman spectra of LIG and PPy-coated LIG were recorded using a 514 nm laser with a Renishaw inVia confocal Raman spectrometer. The electrochemical characterization of LIG electrodes with varying diameters was performed in 5 mM K_4_Fe(CN)_6_/K_3_Fe(CN)_6_ electrolyte. The electrochemical impedance of LIG electrodes was recorded with a three-electrode configuration at frequencies ranging from 0.1 to 10 kHz with an AC voltage of 10 mV using Reference 620+ Potentiostat (Gamry Instruments).

Impedimetric response of a MIP sensor to varying concentrations of cortisol from 0.1 pM to 1 μM in artificial sweat was recorded. The MIP sensor was exposed to interfering molecules in artificial sweat, including progesterone (200 pM), serotonin (10 nM), and cortisone (25 nM), to evaluate the selectivity. The OCP of the sodium ISM-coated electrode was recorded after the sensor was exposed to varying concentrations of NaCl from 10 to 160 mM. For in vivo demonstration, a human subject wore a device to quantify the sweat sodium ion and cortisol concentration at different times of the day, including 9:30 a.m., 1:30 p.m., and 5:30 p.m. All human subject experiments received approval from the Institutional Review Board at Texas A&M University (IRB ID: IRB2019-0811F).

## Supporting Information Available

Optical images of pristine and modified LIG electrodes; contact angle measurements of untreated LIG surface, freshly plasma-treated surface, and one-year-old plasma-treated surface; photographs of wearable devices without plasma treatment for fluid transport; optical images of the paper microfluidic device for wicking rate characterization; iontophoresis-induced and cycling-induced sweat rate measurements; optical images of LIG electrodes with different diameters; 2D Raman peak of LIG fitted with single Lorentzian peak; electrochemical impedance magnitude and phase for the LIG electrodes of varying diameters; electrochemical impedance and electrical resistance of PPy-coated LIG electrodes in a flat position and under bending; OCP stability after exposure to artificial sweat with varying pH (PDF)
