# Supplementary material for: Molecularly Imprinted Wearable Sensor with Paper Microfluidics for Real-Time Sweat Biomarker Analysis
Source: ACS Appl Mater Interfaces. 2024 Aug 23;16(35):46113–22. doi: 10.1021/acsami.4c10033 (PMC11378148; doi:10.1021/acsami.4c10033)
Supplement: Supplementary file 1 — am4c10033_si_001.pdf [file am4c10033_si_001.pdf]

Supporting Information

A molecularly imprinted wearable sensor with paper microfluidics for real-time sweat biomarker analysis

Mayank Garg[a], Heng Guo[a], Ethan Maclam[a], Elizabeth Zhanov[a], Sathwika Samudrala[a], Anton Pavlov[a], Md Saifur Rahman[a], Myeong Namkoong[a], Jennette P. Moreno[c], Limei Tian[a,b]*

[*] Dr. Limei Tian, Corresponding Author
Email: ltian@tamu.edu

[a]Department of Biomedical Engineering, Texas A&M University
College Station, TX 77843, USA.

[b]Center for Remote Health Technologies and Systems
Texas A&M University
College Station, TX 77843, USA.

[c]Department of Pediatrics-Nutrition
Baylor College of Medicine
Houston, TX 77030, USA.

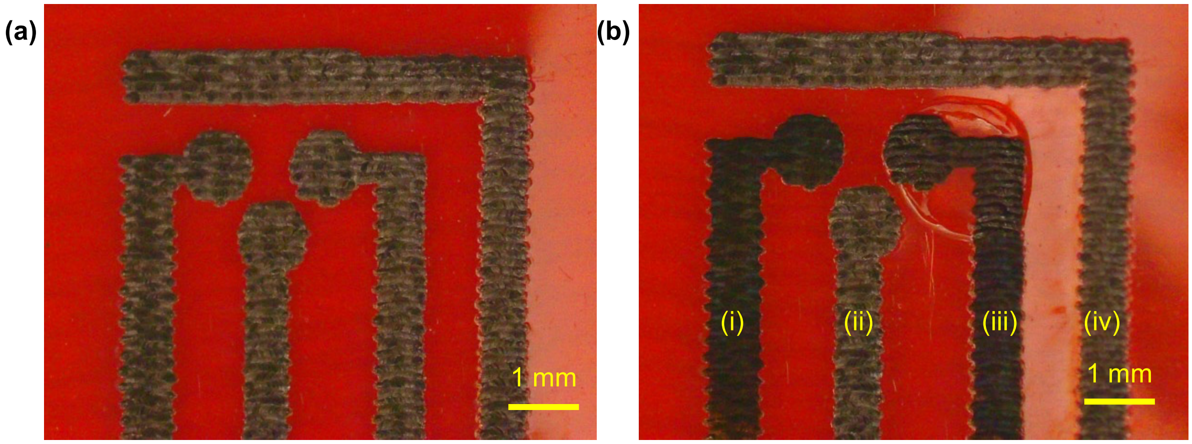

Figure S1. (a) Prinstine and (b) Modified LIG electrodes. LIG electrodes (i) and (iii) were modified with MIP and ISM, respectively. LIG electrode (ii) was modified with Ag/AgCl to work as a reference electrode. LIG electrode (iv) serves as the counter electrode.

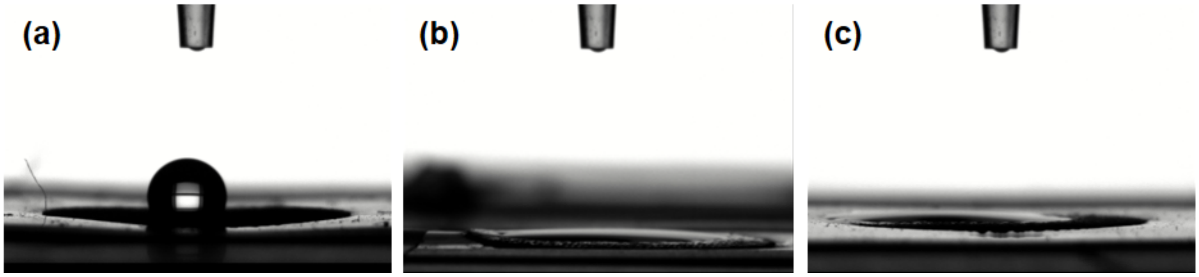

Figure S2. Sessile drop of water on (a) untreated LIG surface, (b) freshly plasma-treated LIG surface, and (c) one-year-old plasma-treated.

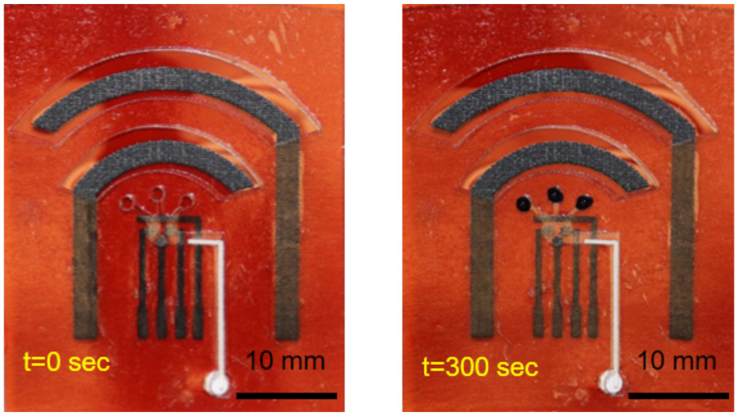

Figure S3. Photographs of the wearable devices without plasma treatment collected at different time points after adding a blue dye solution to the inlet.

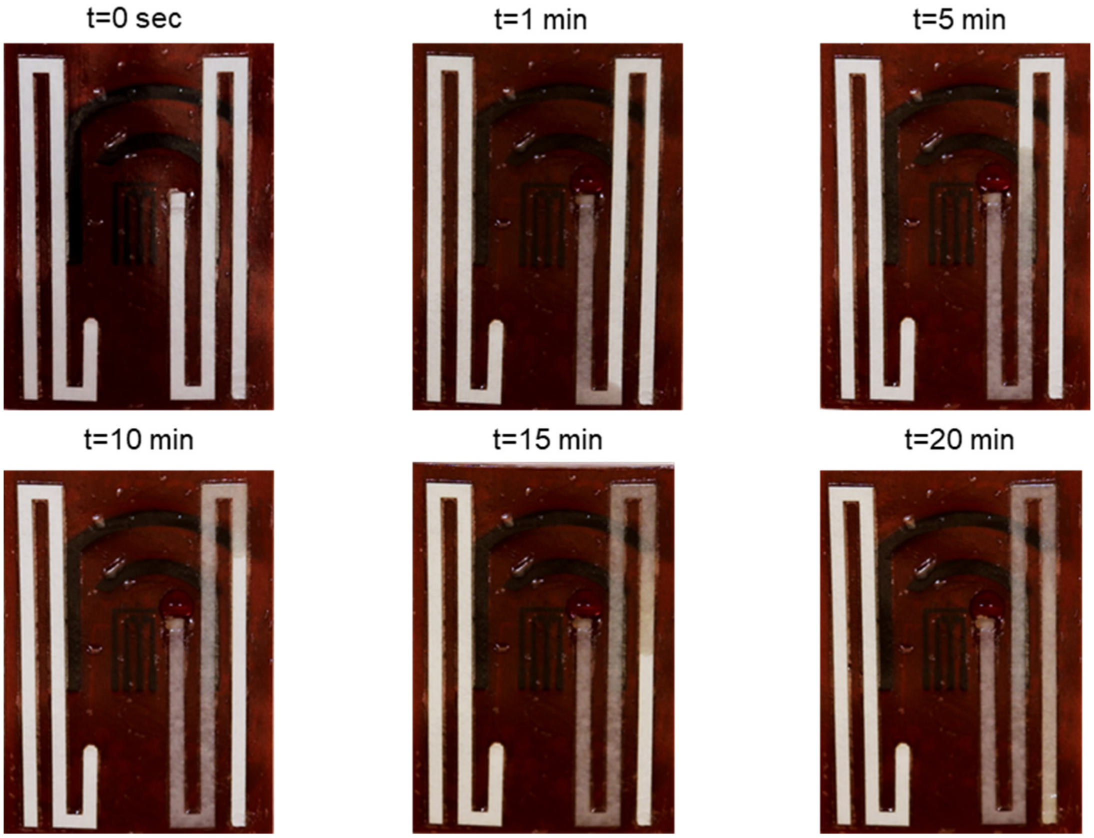

Figure S4. Optical images of the paper microfluidic device acquired at different time points after adding an access amount of fluid to the inlet to quantify the liquid-wicking kinetics.

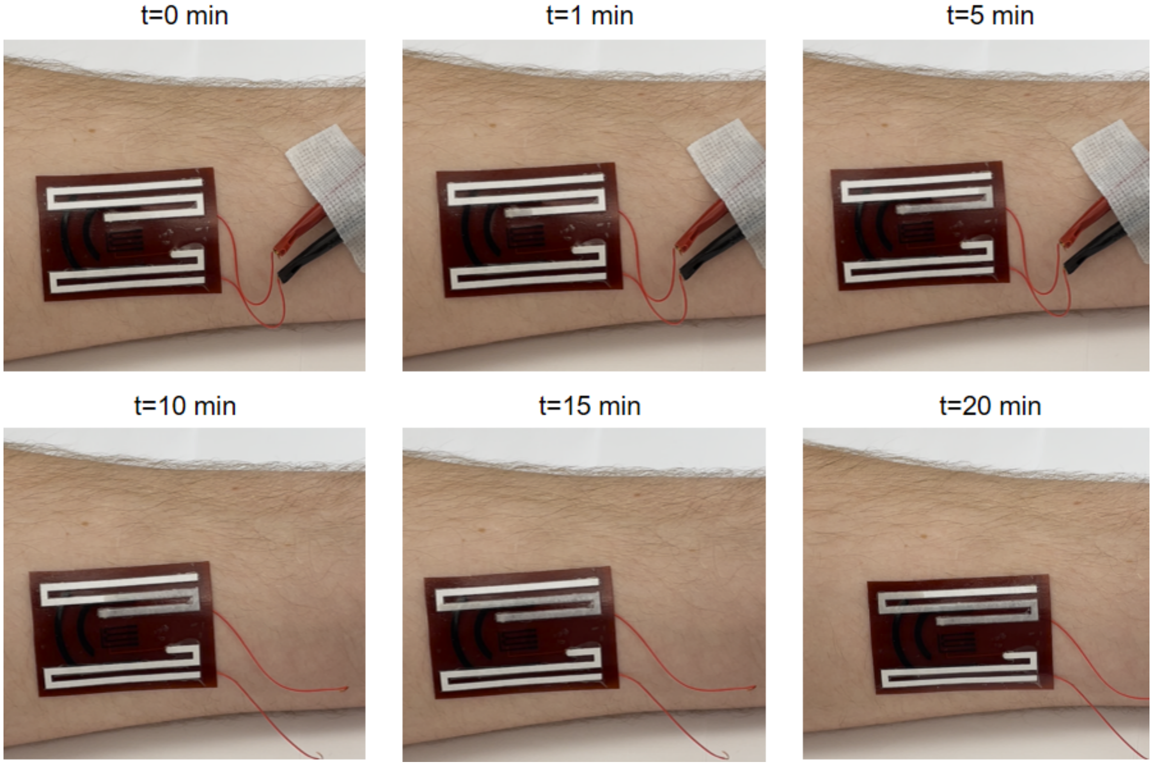

Figure S5. Optical images of the paper microfluidic device applied on the forearm acquired at different time points to quantify iontophoresis-induced sweat rate.

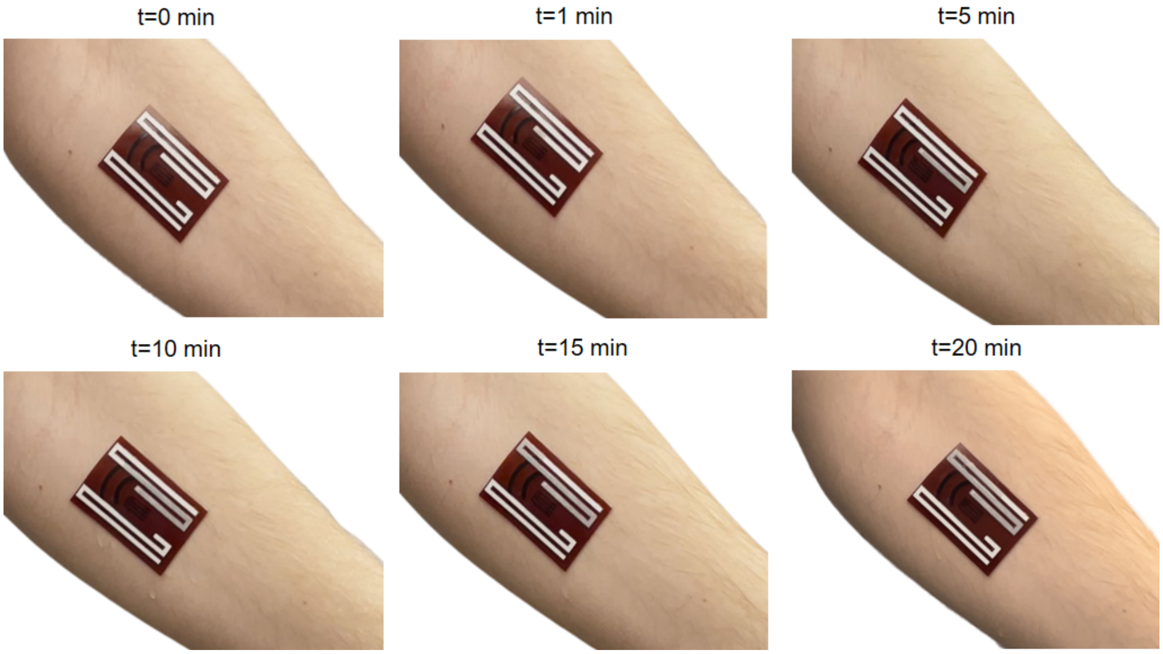

Figure S6. Optical images of the paper microfluidic device applied on the forearm acquired at different time points to quantify the cycling-induced sweat rate.

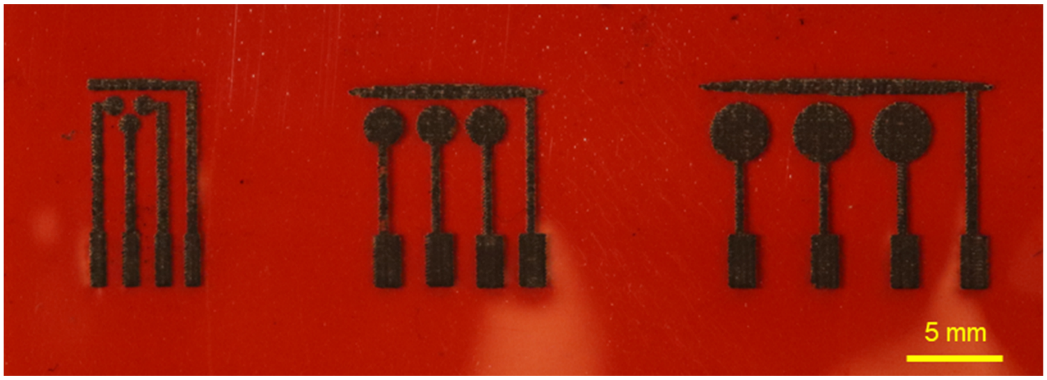

Figure S7. Optical images of LIG electrodes with diameters of 1 mm, 2 mm, and 3 mm from left to right.

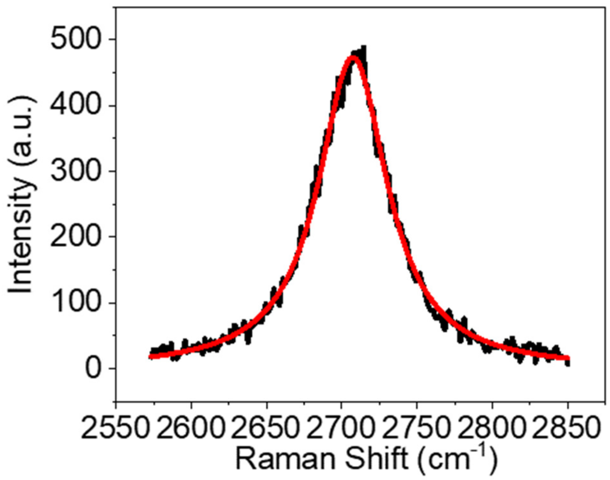

Figure S8. 2D Raman peak of LIG fitted with a single Lorentzian peak.

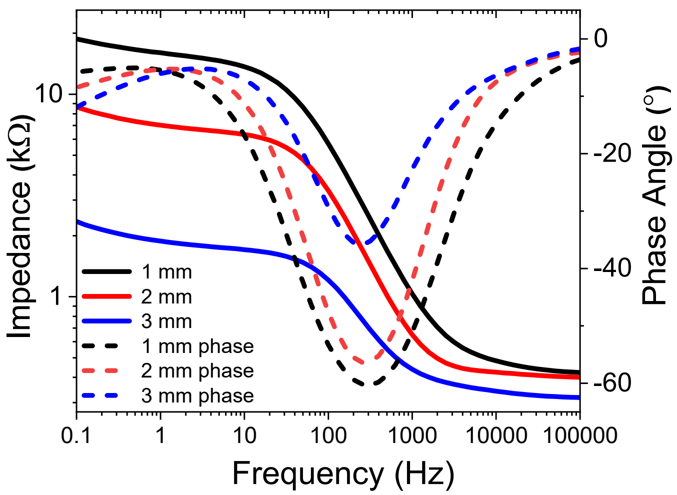

Figure S9. Electrochemical impedance magnitude and phase for the LIG electrodes of varying diameters.

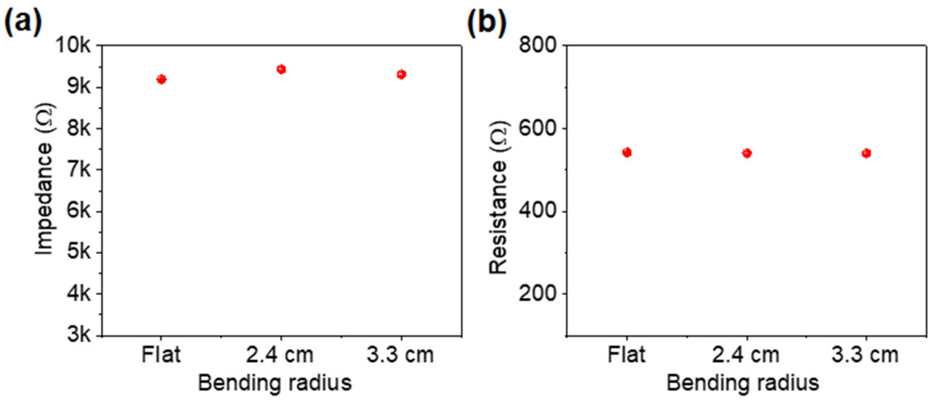

Figure S10. (a) Electrochemical impedance and (b) electrical resistance of PPy-coated LIG electrodes in a flat position and under bending.

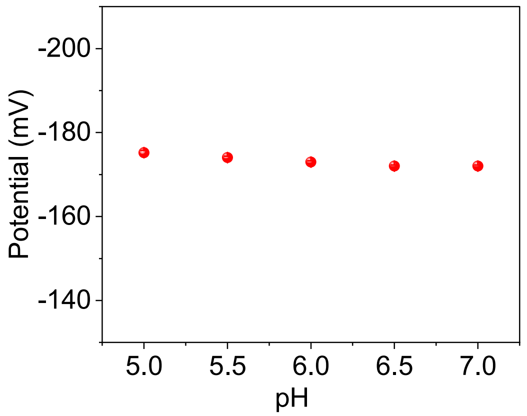

Figure S11. OCP stability after exposure to artificial sweat with varying pH.
